# 1102. Pediatric Hepatitis C Screening by Maternal Hepatitis C Viral Load Status in Pregnancy

**DOI:** 10.1093/ofid/ofad500.075

**Published:** 2023-11-27

**Authors:** Julia DiNicola, Catherine Chappell, Anne-Marie Rick

**Affiliations:** University of Pittsburgh, Pittsburgh, PA; University of Pittsburgh, Pittsburgh, PA; University of Pittsburgh, Pittsburgh, PA

## Abstract

**Background:**

The rise of hepatitis C (HCV) infections among reproductive-age women increases the number of infants at risk for perinatal HCV infection, but screening remains low. It is unknown if maternal HCV viral load (VL) during pregnancy influences infant screening practices.

**Methods:**

Using an existing retrospective cohort of mother-infant pairs with longitudinal well-child care from a single healthcare system, we included infants born from 2015 to 2019 with perinatal HCV exposure based on maternal or infant HCV-specific ICD diagnosis codes and/or laboratory testing and confirmed by reviewing electronic health records. Maternal HCV VL during pregnancy was classified as positive (+), negative (-) or unknown based on HCV RNA testing (or lack-thereof) obtained between 12 months before to 3 months after the infant’s birth date. Adequate infant screening included an HCV RNA test obtained ≥ 2 months of age or an HCV antibody test ≥ 12 months of age. We used univariate logistic regression to compare odds of infant screening based on maternal HCV VL during pregnancy.

**Results:**

Of the 501 HCV-exposed infants, 139 (28%) were born to HCV+/VL- women, 100 (20%) to HCV+/VL unknown women, and 262 (52%) to HCV+/VL+ women. The proportion of screening tests ordered ranged from 68-82%, while test completion ranged from 64-70% (Figure 1). Overall, screening for HCV varied by maternal HCV VL (43% (60/139) HCV+/VL-; 50% (50/100) HCV+/VL unknown; 58% (152/262) HCV+/VL+). Perinatal HCV ranged from 1.7-6% depending on maternal HCV VL status (Figure 1). Infants born to HCV+/VL+ women were two times more likely to have a screening order compared to infants of HCV+/VL- women; but there was no difference for infants of HCV+/VL unknown women (Table 1). Among those with tests ordered, there was no difference in completion between groups (Table 1).

**Figure:**
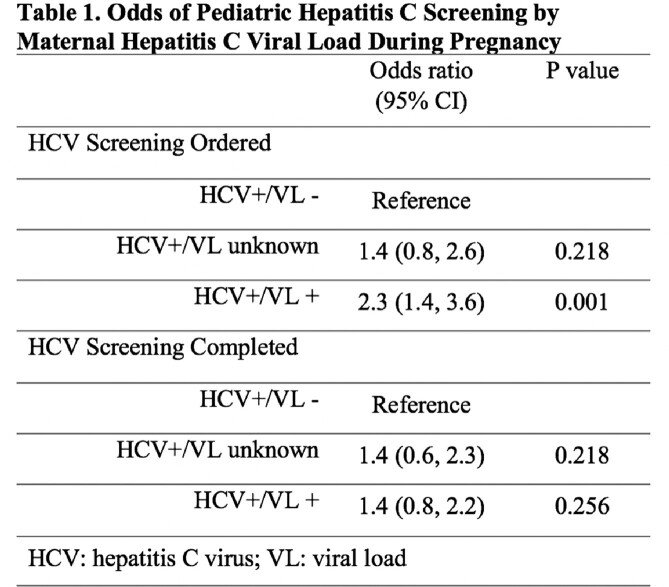

**Figure 1.**
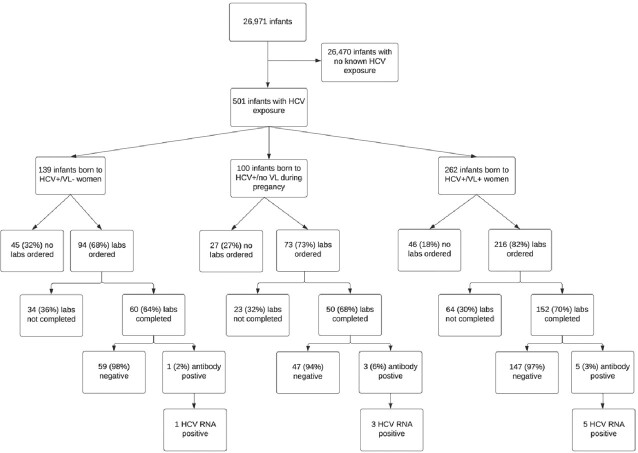
Flow Diagram of Perinatal Hepatitis C Screening of Infants Exposed to Hepatitis C in utero.

**Conclusion:**

Although infants born to HCV+/VL+ women are more likely to be screened for HCV, many, regardless of maternal HCV VL during pregnancy, are never adequately screened and pediatric HCV infections are going undetected. Efforts to increase caregiver and clinician awareness of infant HCV screening guidelines, including consideration of earlier RNA or office-based HCV screening, may improve screening rates and detection of HCV infected infants.

**Disclosures:**

**Catherine Chappell, MD, MSc**, Gilead Sciences: Advisor/Consultant|Gilead Sciences: Grant/Research Support|Organon: Grant/Research Support

